# Supplementary figures and images for: Phage Diving: An Exploration of the Carcharhinid Shark Epidermal Virome
Source: Viruses. 2022 Sep 5;14(9):1969. doi: 10.3390/v14091969 (PMC9500685; doi:10.3390/v14091969)

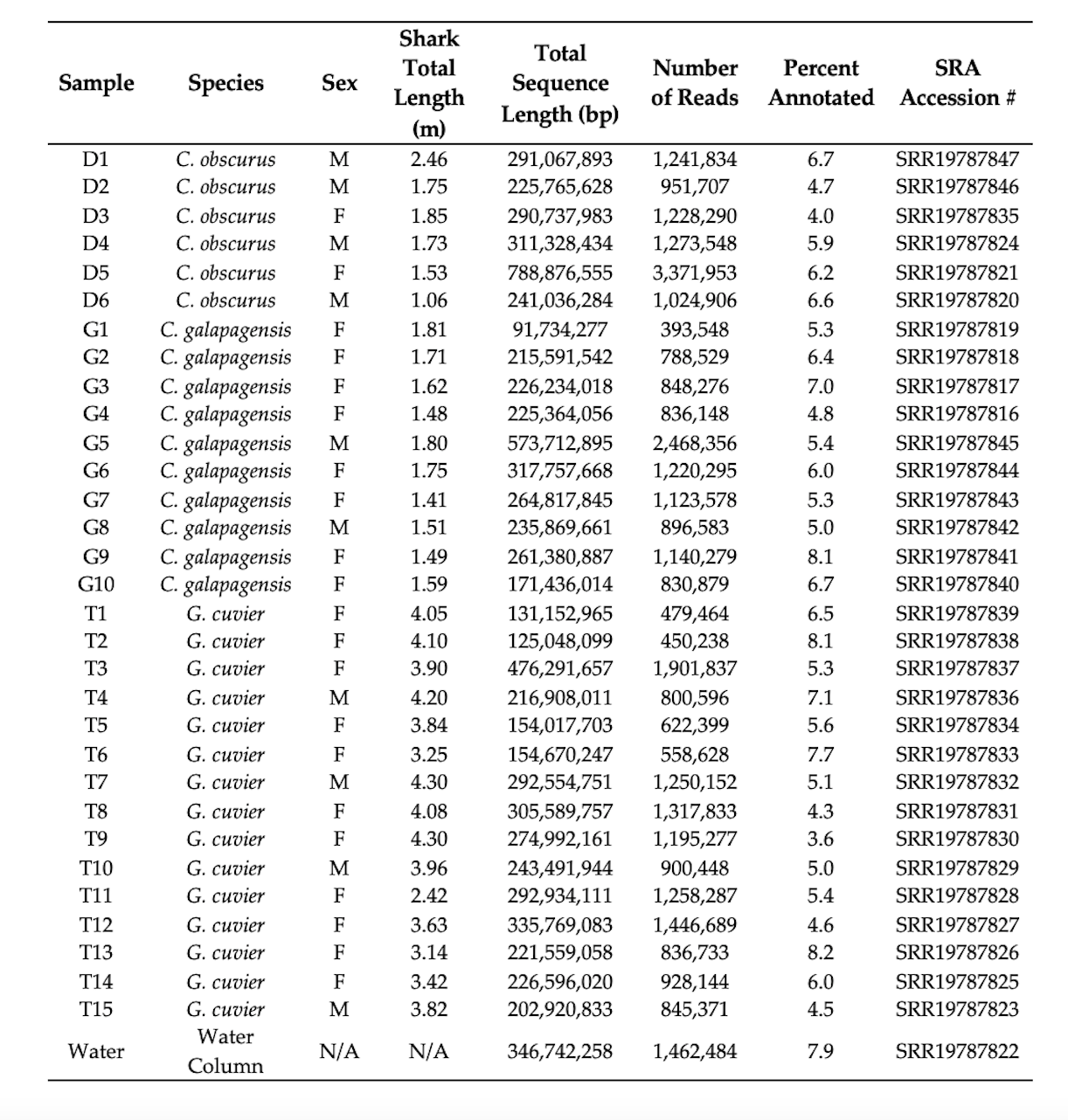

Supplement: Supplementary file 1 [file viruses-14-01969-s001.zip › viruses-1877521-supplementary.png]
